# Fatty Acids Profile and the Relevance of Membranes as the Target of Nutrition-Based Strategies in Atopic Dermatitis: A Narrative Review

**DOI:** 10.3390/nu15173857

**Published:** 2023-09-04

**Authors:** Anna Olejnik, Justyna Gornowicz-Porowska, Dorota Jenerowicz, Adriana Polańska, Małgorzata Dobrzyńska, Juliusz Przysławski, Anna Sansone, Carla Ferreri

**Affiliations:** 1 Faculty of Chemistry, Adam Mickiewicz University in Poznań, Uniwersytetu Poznańskiego 8, 61-614 Poznań, Poland; annamar@amu.edu.pl; 2 Centre for Advanced Technology, Adam Mickiewicz University in Poznań, Uniwersytetu Poznańskiego 10, 61-614 Poznań, Poland; 3 Department and Division of Practical Cosmetology and Skin Diseases Prophylaxis, Poznan University of Medicinal Sciences, Rokietnicka 3, 60-806 Poznań, Poland; 4 Department of Dermatology, Poznan University of Medical Sciences, Przybyszewskiego 49, 60-356 Poznań, Poland; djenerowicz@ump.edu.pl (D.J.); apolanska@ump.edu.pl (A.P.); 5 Department of Bromatology, Poznan University of Medical Sciences, Rokietnica 3, 60-806 Poznań, Poland; mdobrzynska@ump.edu.pl (M.D.); jprzysla@ump.edu.pl (J.P.); 6 Istituto per la Sintesi Organica e la Fotoreattività, Consiglio Nazionale Delle Ricerche, Via Piero Gobetti 101, 40129 Bologna, Italy; anna.sansone@isof.cnr.it

**Keywords:** atopic eczema, membrane remodeling, diet in skin diseases, *n*-3 PUFA, *n*-6 PUFA, membrane fatty acid profile

## Abstract

Recently, the prevalence of atopic dermatitis has increased drastically, especially in urban populations. This multifactorial skin disease is caused by complex interactions between various factors including genetics, environment, lifestyle, and diet. In eczema, apart from using an elimination diet, the adequate content of fatty acids from foods (saturated, monounsaturated, and polyunsaturated fatty acids) plays an important role as an immunomodulatory agent. Different aspects regarding atopic dermatitis include connections between lipid metabolism in atopic dermatitis, with the importance of the MUFA levels, as well as of the omega-6/omega-3 balance that affects the formation of long-chain (C20 eicosanoic and C22 docosaenoic) fatty acids and bioactive lipids from them (such as prostaglandins). Impair/repair of the functioning of epidermal barrier is influenced by these fatty acid levels. The purpose of this review is to drive attention to membrane fatty acid composition and its involvement as the target of fatty acid supplementation. The membrane-targeted strategy indicates the future direction for dermatological research regarding the use of nutritional synergies, in particular using red blood cell fatty acid profiles as a tool for checking the effects of supplementations to reach the target and influence the inflammatory/anti-inflammatory balance of lipid mediators. This knowledge gives the opportunity to develop personalized strategies to create a healthy balance by nutrition with an anti-inflammatory outcome in skin disorders.

## 1. Introduction

**Figure 1:**
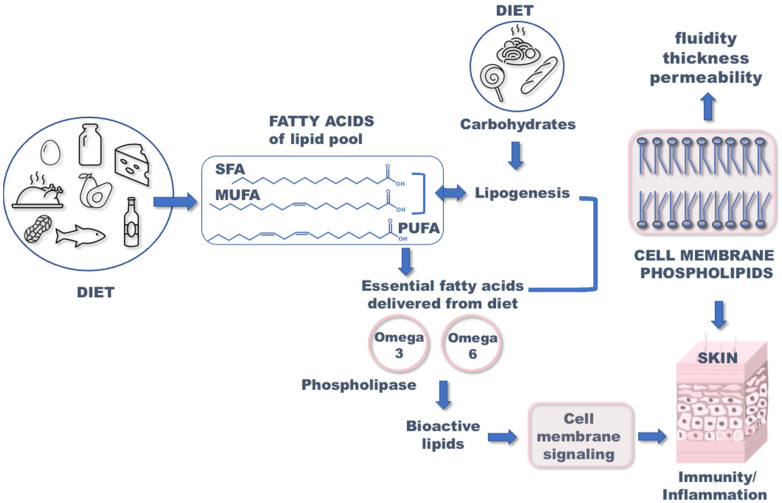
Importance of fatty acids in appropriate functioning of the skin. SFA: saturated fatty acids; MUFA: monounsaturated fatty acid; PUFA: polyunsaturated fatty acid.

Knowledge of cellular mechanisms involved in the onset of atopic dermatitis (AD) can provide significant information to be applied to therapeutical approaches. AD, also known as atopic eczema, is a chronic inflammatory, recurrent, and complex disease characterized by itchy, dry skin [1,2]. Based on current studies, it is assumed that the etiology of atopic dermatitis is related to very complex interactions between various factors including genetics, environment, lifestyle, and diet [3]. Atopic dermatitis can often appear in the first year of life, thus suggesting that the diet of pregnant mothers might influence the appearance of eczema in infants and later in childhood [4,5]. As shown in Figure 1, diet is very important for the delivery of fatty acids (FA) to the human body. Fatty acids are divided into three families: saturated fatty acids (SFA), monounsaturated fatty acids (MUFA), and polyunsaturated fatty acids (PUFA), which are structural and functional components of cell membrane phospholipids. The appropriate combination of these three structurally diverse molecules is specific for each tissue and also for skin [6]. For fibroblasts and keratinocytes (two major cell types present in skin tissues), such compositions are known to be influenced by adding supplemental fatty acids that was proved in cell culture studies [7]. The alteration of fatty acid composition affects the biophysical properties of membranes such as fluidity, thickness, and permeability, which have implications on cell function [8]. Consequently, the composition of FA in cell membranes has an impact on hydration, resistance, and skin aging [9]. It was proved that essential fatty acids (EFA) play a significant role in retaining the proper structure of the epidermal cell membrane [10].

**Figure 2:**
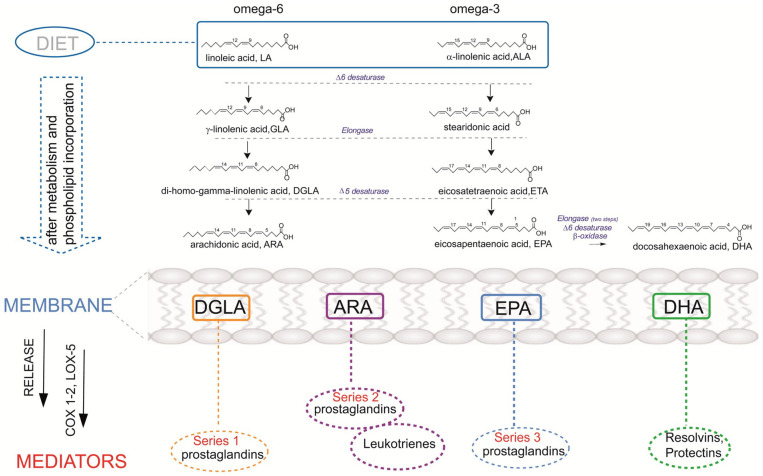
The diet–metabolism cascades providing omega-6 and omega-3 polyunsaturated fatty acid (PUFA) for membrane phospholipids and some lipid mediators obtained after the release of PUFA from membranes. COX: cyclooxygenase enzyme, LOX: lipoxygenase enzyme.

Thus, the deficiency of EFA is known to affect skin conditions [11] and the impaired function of the skin barrier may be related to the onset of atopic dermatitis. The transformation of SFA into MUFA is regulated by desaturase enzymes (stearoyl-CoA desaturase) [12], which is a crucial step for fibroblast growth. Moreover, PUFA (omega-6 linoleic acid C18:2 and omega-3 alpha-linolenic acid C18:3) are introduced exclusively through diet, activating the enzymatic cascades to produce long-chain (C20 eicosanoic and C22 docosaenoic) fatty acids. PUFA molecules are incorporated in membrane phospholipids and liberated by phospholipase enzymes to produce bioactive lipid (BAL) mediators (Figure 2). They contribute to physiological functions and are involved in pathological states as well as the onset and resolution of inflammation processes. Since skin is characterized by bioactive lipid metabolism, FAs are of great importance considering structural and functional integrity, particularly when converted into bioactive mediators. BAL exhibit a high impact on skin responses either in allergic or oxidative processes accompanying diseases [13,14,15]. PUFA are precursors of bioactive mediators, including eicosanoids, as shown in Figure 2 and further explained in this review. It was suggested by Miles et al. [5] that eicosanoids as oxygenated metabolites of omega-6 PUFA arachidonic acid promote allergic inflammation, and the intake of omega-3 such as eicosapentaenoic acid (EPA) and docosahexaenoic acid (DHA) may oppose this action through the corresponding BALs. The status of pregnant women regarding PUFA has been addressed with fish oil supplementation as a strategy to prevent atopic and allergic diseases, but results are contrasting. However, only fish oil cannot contribute to the balance between the two PUFA metabolic cascades (omega-6 and omega-3) and it is necessary to understand at which step of one or the other pathways the unbalance/impairment occurs. In particular, regarding the role of DGLA (dihomo gamma linolenic acid), which is an omega-6, DGLA generates bioactive lipids (prostaglandin series 1) with anti-inflammatory activity (Figure 2), thus balancing the activity of BAL derived from arachidonic acid [8,9]. Therefore, lipidome analysis with a focus on the fatty acid composition is crucial information to acquire in order to understand the causes of an effect on the skin properties and reactivity, such as epidermal barrier impairment and itching. It is often said that diet can be reflected in skin conditions; however, a more detailed scenario can be achieved by knowing, in particular, the nutritional and metabolic status of the subjects and the resulting membrane fatty acid composition. 

In this review, the recent aspects concerning AD are summarized, including epidemiology, genetics, and diet. Particular attention is given to the association of fatty acids profile with atopic dermatitis. 

## 2. Methods

We conducted a review of the literature to provide an overview of the recent data concerning the role of fatty acids profiles in atopic dermatitis. The results are presented in the form of a topical review. Searches were conducted using the following databases: PubMed and the Google Scholar. Multiple search terms were used, including “atopic dermatitis”, “fatty acids”, “diet”, and “nutrition”. Only English language papers published in peer-reviewed journals and book chapters were included. Initially, abstracts were read to assess their relevance. Using this method, 150 papers were identified. Each was subsequently read and analyzed. This led to the identification of further relevant references, and an additional 107 relevant papers were included in this review.

## 3. Epidemiology of Atopic Dermatitis

Atopic dermatitis is a chronic, inflammatory skin condition. The causes of such an increase are likely the changes in the environmental conditions, due to growing industrialization and environmental pollution, playing roles in the onset of the disease as well as in its course [16,17]. However, it should be emphasized that an increase in AD prevalence may also be associated with an increased ability for both doctors and parents to recognize the disease, together with the development of new, more objective diagnostic methods. It was also explained that stricter hygiene and lack of contact with bacterial antigens and allergens in childhood later favors the development of an abnormal immune system reaction to common environmental factors [18,19]. 

Early onset AD usually appears in babies or during childhood and the highest incidence of AD is observed during infancy, and disease onset takes place by the age of 7 years [20]. It is clinically characterized by exacerbations and remissions of eczematous pruritic skin lesions with scaling, dry skin, and susceptibility to cutaneous bacterial and mycotic infections [21,22]. During recent decades, there has been a significant increase in the incidence of allergic diseases, including atopic dermatitis. 

Validated data on the frequency of eczema are based on a standardized methodology in ISAAC (International Study of Asthma and Allergies in Childhood). In 90 centers, 256,410 children aged 6–7 years, and at 151 locations, a total of 458,623 children aged 13–14 years were investigated in 56 countries. ISAAC confirmed a high worldwide variation in the disease, for younger children ranging between 1.1% in Iran and 16% in Japan and Sweden. For children 13–14 years old, the prevalence ranges between 1% in Albania and 17% in Nigeria. According to this study, prevalence seemed to be higher in Australia and Northern Europe and lower in Asia and Central and Eastern Europe. Updated prevalence and incidence data considering AD suggest a stabilized level in Europe and North America and, in contrast, an increase in other continents (i.e., Asia). In children, the point prevalence of AD is estimated from 0% up to 18.2% [23,24]. There are many fewer studies evaluating the frequency of AD in adults (by questionnaire or clinical examination). The data presenting the prevalence of atopic dermatitis in adults in different countries are collected and presented in Table 1. 

In comparison to worldwide trends, European studies imply similarities: AD is more prevalent in children compared to adults and in overpopulated cities. The prevalence in the adolescent group is between 1.5% (Lithuania) and 15% (Bulgaria, Denmark, Finland, and Hungary). It seems that miscellaneous factors (genes, culture, climate, economy) may influence these differences [24,30]. Concerning patients’ gender, both the 1-year prevalence and lifetime prevalence of diagnosed AD seem to be higher in females than in males. The only exceptions are the UK (prevalence is same—2.5%) and the USA (prevalence estimated as higher in males—5.1% vs. 4.6%—not significant). Contributing factors to AD onset currently include dysbiosis, systemic immune responses, and neuroinflammation, the last is involved in itching [31].

## 4. Genetic Aspects 

The pathogenesis of atopic dermatitis is still unclear. However, it is generally known that it results from dysfunction of the immune response and skin barrier. Apart from environmental factors, genetics may be also associated with AD. Recent studies proved that AD was influenced by genetic alterations. Furthermore, the interactions of many different genes (not only one single gene) play a significant role in the progression of dermatosis. Investigation on twins has shown a role in genetic background, with a concordance rate of 72–86% in monozygotic twins and 21–23% in dizygotic twins, demonstrating high heritability of AD [32,33]. Due to the development in molecular biology, different gene candidates in atopic dermatitis (over 70) have been identified [34]. They can be divided into five main important groups depending on the pathological process in AD [35]. The first group represents genes and mutations that lead to dysfunction in the epidermal barrier, such as filaggrin (FLG). The mutations of FLG (located in the region known as the epidermal differentiation complex) were linked with atopic dermatitis by various research groups that carried out studies on American, European, and Asian populations [36,37,38]. FLG is responsible for encoding a structural protein crucial in the formation of the skin barrier [39]. The second group includes genes of the innate immune mechanism that cause the over-reactivity of the TLR system (TLR1, TLR2, TLR4, TLR6, TLR9, TLR1). The third group comprises genes that are related to adaptive immune response mechanisms (genes of Th2 response—IL4, IL5, IL13, IL2RA, IL-13RA). The fourth group contains genes encoding alarmins formed by keratinocytes (interleukin IL-25, IL-33, TSLP). The last is represented by genes that regulate the vitamin D pathways (CYP27A1, CYP2R1, VDR) [40,41]. Importantly, PUFA composition is strongly controlled by the gene encoding the fatty acid desaturases (FADS) cluster [42,43]. Polymorphisms in the FADS genes influence the contribution of PUFAs and LC-PUFAs to total lipids [44]. The conversion of essential fatty acids to longer-chain, biologically active metabolites is regulated by the ∆5 and ∆6 desaturase enzymes, which are encoded by the FADS1 and FADS2 (see Figure 2). Therefore, it is hypothesized that interindividual genetic differences may modify the association between dietary fatty acid intake and AD or lipids [45,46,47,48]. Most of these polymorphisms have adverse effects and cause lower blood levels of DGLA, DHA, EPA, and arachidonic acid (AA). The association between SNPs in the FADS gene cluster and atopy has been reported in German studies (LISA—“Influences of Lifestyle-related Factors on the Immune System and the Development of Allergies in Childhood”, GINI—“The German Infant Nutritional Intervention Study”) and a Dutch study (KOALA—“Kind, Ouders en gezondheid: Aandacht voor Leefstijl en Aanleg”). Inconsistent results were obtained: LISA indicated that SNPs is significantly associated with eczema, but the KOALA cohort contradicts this thesis [43,49,50,51]. Chisaguano et al. [33] revealed that lower mRNA expressions of FADS2 and elongase enzymes, ELOVL5, are associated with a higher risk of atopic eczema in children. AD is associated with lower ELOVL5 mRNA levels than in controls, which may explain the lower levels of DGLA and AA in AD (elongase-5 participates in the biosynthesis of these FA). Barman et al. [52] documented that the presence of rs102275 and rs174448 haplotypes had lower levels of AA, and increased levels of DGLA, which significantly reduce the risk of atopic eczema. This study indicates the important role of LC-PUFA in AD development and showed that limited activity of desaturases reduces this risk. 

## 5. Molecular Aspects

It was reported that the skin of patients with AD is characterized by a deficiency in ceramides and antimicrobial peptides, which protect from infectious agents [39]. These shortages lead to transepidermal water loss and enhancement of allergen penetration into the skin. Moreover, the skin of patients is very often colonized by *Staphylococcus aureus*, which promotes the pathology [53]. The interplay of various factors causes the responses of T-cells in the skin with the subsequent release of cytokines and proinflammatory chemokines, which promote the production of immunoglobulin E (IgE) and systemic inflammatory reactions, leading to skin itching [54]. In turn, the precise molecular mechanism underlying the association between dyslipidemia and AD is contrasting. Some of the studies concluded that AD is associated with hyperlipidemia, but others do not confirm this observation. Recent data have indicated that dyslipidemia is related to an altered adaptive immune response of Th2 cells, which may lead to AD development [32]. The presence of a chronic inflammatory state in dyslipidemia is likely responsible for long-term skin inflammation and provides a possible mechanism for the relationship between AD and hyperlipidemia [33]. In particular, elevated triglycerides (TG) and low-density lipoprotein cholesterol (LDL-C) lead to increased proinflammatory signaling with elevated levels of cytokines TNF-α and interleukin (IL)-6 [55]. In contrast, high-density lipoprotein cholesterol (HDL-C) has an anti-inflammatory effect [55]. Accordingly, an association of hyperlipidemia with the severity of AD was proved by Kim et al. [32], who revealed that the SCORAD score, a clinical tool used to assess the extent and severity of eczema, has positive associations with TG and a negative association with HDL-C. The combined intakes or supplementation of omega-6, gamma-linolenic acid (GLA), and omega-3 long-chain polyunsaturated fatty acids (LCPUFAs) exhibit the highest potential in diminishing inflammatory processes, which could be beneficial for the management of AD [56]. 

## 6. Diet and Atopic Dermatitis 

According to current literature, diet may influence the immune system and prevent disease [57,58]. In eczema, apart from using an elimination diet, the adequate content of FA plays an important role [17,57]. Clinical observations and experimental studies indicate that serum fatty acid composition in atopic dermatitis patients is often imbalanced [59]. It is supposed that a balanced diet, rich in omega-3 PUFAs, the proper balance of omega-3/omega-6, and reduction in saturated fatty acids (SFA) and trans fatty acids (TFA), may diminish inflammation and improve skin condition. The main food sources of FA related to AD are presented in Table 2. 

Depending on chemical structure and biochemical properties, fatty acids can be divided into saturated, monounsaturated, and polyunsaturated families (SFA, MUFA, and PUFA). The SFA are carboxylic acids that have a typical linear molecular structure with no double bonds between carbon atoms (Figure 1). The most common SFA in the human body, and the first to be metabolically produced, is palmitic acid (C16:0), constituting 28–32% of total fatty acid content in serum [61]. Food sources of SFA are meat, dairy products, and highly processed food. In the study by Hoppu U. et al., an association between higher content of SFA in maternal diet and higher atopic sensitization of infants was observed [62]. In the International Study of Asthma and Allergies in Childhood, consumption of fast food (rich in SFAs) three times per week or more was linked to the development of AD [63,64]. The role of SFA in atopy is not fully understood and requires further studies. 

### 6.1. Effect of Monounsaturated Fatty Acids on Atopic Dermatitis

MUFAs are characterized by one double bond in the carbon chain (Figure 1). The main sources of MUFA oleic acid (C18:1) in the human diet are vegetable oils, especially olive oil and high-fat fruits, like avocados or olives. Some studies have shown that atopic lesions contained an increased content of MUFA in phosphoglycerides, compared to lesion-free epidermis [65]. Moreover, subsequent studies suggested that a high intake of MUFAs may lead to the development of atopic disease [66,67]. Actually, molecular studies on the effects of MUFA on the skin barrier using an in vitro model showed that an increment of these fatty acids causes reduction in the lipid barrier in the stratum corneum [68]. Since MUFA is present in the ceramide structures as principal components of sebum, the alteration of such fatty acids was largely studied in this type of specimen, also for its easiness of withdrawal from patients. Sebocytes have their own lipid biosynthesis and contribute to the stability of the skin barrier, and the studies available so far underline the importance of performing combined molecular and clinical investigations [69]. It is supposed that the negative effect of MUFA on atopy may also be related to the simultaneous reduction in the omega-6 and omega-3 PUFAs in the diet. It is worth noting that MUFA are also the only unsaturated fatty acids that can be metabolically produced, which occurs by desaturase enzymes working on SFA. To complete the scenario of the biologically active fatty acids, PUFAs are important components that must be introduced by the diet, since essential fatty acids are not synthesized by human cells. Therefore, the enrichment of MUFA in the skin can be interpreted as the metabolic response to increase unsaturation content, when the polyunsaturated precursors, omega-6 linoleic acid and omega-3 alpha-linolenic acid, are deficient or less present in the diet.

### 6.2. Effect of Polyunsaturated Fatty Acids on Atopic Dermatitis

Indeed, PUFAs belong to the group of FA with 18 or more carbon atoms; they contain at least two double bonds between carbon atoms. There are two families of PUFAs: omega-3 and omega-6, depending on the position of the final double bond. PUFAs play the role of assuring the correct environment for membrane protein function, maintaining membrane fluidity, and regulating cell signaling, cellular function, and gene expression [70]. The deficiency of PUFAs may contribute to the development and manifestations of atopy. It is assumed that excessive intake of omega-6 PUFA is related to risk factors for eczema, while omega-3 PUFAs have the opposite effect [71]. The increase in the incidence of atopic dermatitis is attributed to the imbalance in dietary components [72], in particular for the lipid intakes the higher consumption of omega-6 fatty acids compared to omega-3 can play a role, as previously mentioned [56,66]. Horrobin reported the changes in PUFA profiles in patients suffering from AD [59]. In particular, underlining the importance of delta-6 desturase enzyme, GLA (gamma linolenic acid) was individuated as a relevant element of skin cell structure, and a crucial precursor of DGLA to generate anti-inflammatory lipid mediators (see Figure 2), Therefore, dietary sources, in the form of oils such as borage or primrose oils, became important as a unique source of GLA in the diet, to be used for targeted supplementations. As far as indirect effects on MUFA levels are concerned, Schäfer et al. observed that the supplementation of primrose oil rich in PUFA omega-6 (20–40% content of GLA) increases the omega-6/MUFA ratio in the lesioned epidermis, which contributes to the improvement in the skin condition [73]. Children with atopic dermatitis showed similar impairment of the membrane fatty acid profiles in erythrocytes and T-lymphocytes [9], in particular also involving the geometry of the unsaturated fatty acids, which is naturally cis and can be transformed into trans under conditions of free radical and oxidative stress [74]. 

### 6.3. Different Dietary Sources of Omega-6 and Omega-3

The PUFA omega-6 and omega-3 pathways with their insertion in membrane phospholipids and production of eicosanoid mediators are shown in Figure 2 [75]. The most common omega-6 PUFA in foods is linoleic acid (LA, C18:2). It is found in vegetable oils like sunflower, corn, and safflower oil [60]. In recent decades, it was observed that the Western diet, with increased consumption of red meats and refined vegetable oils, is characterized by low content of omega-3 PUFAs and increased levels of omega-6 PUFAs and SFA contribute to the development of eczema [3,63,64,65]. Increased intake of omega-6 PUFAs in a diet, especially LA, activates the omega-6 enzymatic cascade that leads to an increased synthesis of the long-chain PUFA arachidonic acid (AA, C20:4) (see Figure 2). Such enrichment in the fatty acid precursor is reflected by the increased level of AA in the cell membrane phospholipids, thereby creating the premise for an increased detachment of AA from membranes with subsequent production of eicosanoid mediators [76]. Such mediators derived from AA are prostaglandins of group 2 (PGD2, PGE2, PGF2, PGI2), thromboxanes, leukotrienes, and lipoxins [77]. Direct sources of AA are also foods like meat and eggs [78]. Some studies suggest balancing a typically LA- and AA-rich diet with gamma-linolenic acid (GLA, C18:3, *n*-6) and omega-3 fatty acids, which can act to balance AA, as the molecular content of membrane phospholipids in DGLA and/or inhibit the production of proinflammatory eicosanoids (see Figure 2). As already noted in Section 6.2, natural sources of GLA are vegetable oils like evening primrose, borage, and hempseed oils (Table 1). It is worth noting that GLA-rich oils used in 12 clinical trials of oral or topical borage oil for the treatment of atopic dermatitis and one preventive trial are reported to have a small effect on eczema [79]. Here, we can comment that GLA is also the precursor of AA in the omega-6 pathway (see Figure 2); therefore, the effect of GLA supplementation must be carried out after knowing the balance between the omega-6 and omega-3 molecular contents in the subject. Indeed, it was already shown that it is necessary to add omega-3 alpha-linolenic acid to the GLA supplementation to overcome arachidonic acid accumulation [80]. Previous studies suggested that GLA, dihomo-γ-linolenic (DGLA), and arachidonic acids, present in epidermal phospholipids, are biosynthesized endogenously elsewhere and transported in the bloodstream to the epidermis, where phospholipid biosynthesis takes place [33]. Lower DGLA blood levels were observed in AD than in controls [33]. Interestingly, Kawashima et al. [78] reported that oral administration of DGLA effectively prevents the development of AD in mice models. In light of this, the possible molecular mechanism for regulating the prostaglandin D1 (PGD1) supply was proposed by Amagai et al. [81].

Omega-3 PUFAs are known to play beneficial effects on atopic dermatitis by several mechanisms, such as:

(1) Competition on the enzymatic cascade by omega-3 PUFAs that inhibits the omega-6 AA formation and diminishes the contribution to PGE2 formation (Figure 1);
(2) Direct activity of omega-3 and their complexes with peroxisome proliferator-activated receptors to create a ligand-activated transcription factor of anti-inflammatory genes, influencing inflammatory and immunologic responses [82,83].

Foods rich in omega-3 PUFAs include fatty fish, algae, flax seeds and oil, chia seeds, and walnuts (Table 1) [57]. In the omega-3 cascade, alpha-linolenic acid (ALA, 18:3) from plants is the precursor, which must be introduced from the diet; subsequent enzymatic transformations produce long-chain omega-3 PUFA (LCPUFA), namely DHA (22:6) and EPA (20:5), which are formed with different capability in the human body (Figure 2). It is worth underlining that for up to 6 months of life, babies have diversified activities of desaturase enzymes and unbalances between omega-6 and omega-3 [84]. This is a significant point for AD in infants for two reasons: (1) it becomes important to provide a balanced intake of essential fatty acids with their food, principally milk, with breast-feeding the best source with attention to mothers’ diet; (2) the study of the fatty acid balance in each individual should become a tool for the personalization of the lipidome status in dermatological problems [9]. 

EPA and DHA also come from marine sources, and these food intakes must be carefully inquired in dermatological patients to individuate the effects in their diets. In lesional atopic epidermis, a low content of long-chain PUFAs was observed. There are many randomized clinical studies that analyzed the supplementation of fish oil, rich in omega-3 LC-PUFAs, in atopic patients. Results have shown some benefits to the severity of eczema. ALA supplementation can increase omega-3 fatty acids in the blood. However, there are too few studies confirming the effect of its use on the improvement in skin lesions. More recent studies presented the composition of eicosanoids (molecules formed by oxidation of PUFAs) in the blood of patients with atopic dermatitis. A notable decrease in docosahexaenoic acid, leukotriene B_5_, and lipoxin A_4_ and no increase in linoleic acid was observed [85], unlike previous studies [86]. Furthermore, it was also reported that the levels of 5-hydroxy-eicosapentaenoic acid EPA slightly declined in AD patients [85]. In turn, Barman et al. [3] studied if there is a relationship between the proportions of FA in infant and maternal plasma phospholipids and the incidence of atopic dermatitis during the first year of life. The correlations between the fatty acid profiles were also associated with the mother’s diet during pregnancy. It was revealed that higher proportions of omega-6 PUFAs and lower amounts of omega-3 PUFAs in newborn phospholipids were related to the elevated risk of atopic dermatitis development in the first year of life. The level of omega-3 PUFA increases with higher fish consumption, while the proportion of *n*-6 PUFA rises with a greater intake of meat. These studies demonstrated that supplementation of fish oil during pregnancy might reduce the severity of AD in childhood. 

### 6.4. Effect of Trans Fatty Acids on Atopic Dermatitis 

**Figure 3:**
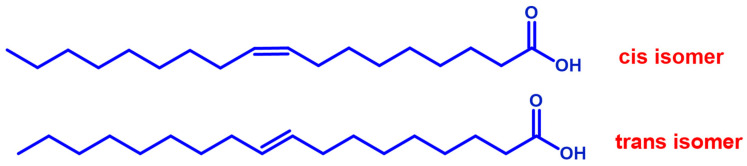
Geometrical isomers of a representative monounsaturated fatty acid.

Trans fatty acids (TFA) are unsaturated fats with trans double carbon bonds instead of cis bonds (Figure 3). Some nutritional studies have suggested the involvement of TFA contained in meat, milk, margarine, or partially hydrogenated fats intake in allergy disease and eczema intensification [87,88]. Conversely, transgeometrical isomers of fatty acids are unnatural lipids that have the same position of the double bond found in the cis counterparts, but with the opposite geometry. These TFAs are formed as the result of free radical reactivity toward unsaturated lipids and have been individuated in children with atopic eczema/dermatitis syndrome. They are significantly higher in both red blood cells and T-cell membrane phospholipids [89].

The endogenous and exogenous sources of TFA provide an important indication of exposure to either cellular stress or dietary factors, respectively, which are discussed for several diseases [90]. In dermatology, TFAs might affect the metabolism of fatty acids, contributing to the alteration of the fatty acid profile [91]. TFA may inhibit desaturation activity in human skin fibroblasts [92,93]. TFA intake is associated with higher tumor necrosis factor receptor levels [94]. Interestingly, the proinflammatory effects of TFA may be stronger for trans-linoleic acid and trans-oleic acid rather than trans-palmitoleic acid, but further study is needed [95]. A summary of some evidences discussed in this review is provided in Table 3, mainly concerning atopic disease. 

Currently, there are no strict recommendations regarding fat consumption and the proportion of fatty acids in the diet for atopic dermatitis patients. A properly balanced diet with a low SFA and TFA content and an appropriate ratio of omega-3 to omega-6 and MUFA to PUFA emerge to be the proper treatment direction. Appropriate approaches combining diet and supplementation require further epidemiological and clinical studies, but a paradigm change is also needed toward the introduction of such molecular analysis in panels for diagnosis and treatment. A recent paper suggests that omega-3 fatty acids are relevant in screening and treatment of atopic dermatitis risk, remarking once more the need for serious consideration from the medical community [96]. A review on a total of 38 studies reported benefits for omega-3 fatty acid supplementation in the treatment of psoriasis, atopic dermatitis, acne, and skin ulcers [97]. When supplementation is carried out, the detection of the PUFA levels in patients before and after supplementation is an important element to discriminate the molecular effects on the causes of the disease. 

## 7. Fatty Acid-Based Membrane Lipidomics in Atopic Dermatitis

Based on the fatty acid scenario depicted so far, it is evident that fatty acid-based membrane lipidomic analysis can offer a tool for both medicinal and biological research and clinical development in dermatology. It enables check-up of molecular unbalances and the absence of essential elements, such as PUFA and other fatty acids, such as SFA and MUFA, and their trans isomers, with known activities on membrane permeability and fluidity, cell signaling, and processes. By knowing the membrane lipidomic profile of the subject, it is possible to develop a personalized strategy, called membrane lipid therapy [98], for restoring and maintaining a healthy balance, and, in particular, creating a fatty acid balance for the correct functioning of the inflammatory-resolution response. It should be noted that FA can also influence skin permeability [3]. The analysis of fatty acids in red blood cell (RBC) membranes involves several steps, including the initial membrane isolation and phospholipids extraction, followed by transesterification of fatty acids to FAME (fatty acid methyl esters) and their qualitative and quantitative determination by gas chromatography [99]. This analysis is relevant in dermatology since inflammation affects a variety of skin problems, and it is necessary to determine the levels of C20-C22 fatty acids, such as omega-6 arachidonic acid, DGLA, omega-3 EPA, and DHA, all of which are precursors to lipid mediators involved in this response (Figure 2). It should be noted that fatty acid profiles are also considered in whole blood and plasma samples [55,100]. However, the meaning of fatty acids is completely different depending on the type of sample; the analysis performed in plasma provides information about fatty acid contents in circulating lipids, strongly influenced by short-term dietary consumption, and by the few days before blood sampling. In turn, RBC membrane fatty acids provide more stabilized information obtained from the combination of nutritional and metabolic transformations. It is worth recalling that the fatty acid pool present in the body is used to form phospholipids for all cells during tissue growth or turnover, and 80% of the cells created daily is RBC [101]. This renders RBC a representative cell for the fatty acid pool and for examination of its variations due to nutritional and metabolic conditions. Undoubtedly, analysis of fatty acids in RBC membranes requires more steps of cell and membrane isolation, followed by careful chemical transformation conditions to obtain the fatty acid residues from membrane phospholipids, as previously explained. Nevertheless, due to the role of cell membranes in the release of bioactive mediators, as previously explained and depicted in Figure 2, it was underlined that shortcuts to obtain fatty acid levels from less significant compartments are not appropriate for precision medical applications [102]. The shape of RBC is maintained by the appropriate ratio of the outer and inner lipid bilayers, the ratio of cholesterol to phospholipids, the ratio of phosphatidylcholine to sphingomyelin, and the amount and type of fatty acids [103]. The excessive rigidity of cell membranes and, consequently, impaired ability to deformation, are caused mainly by changes in the lipid and fatty acid composition [9,104]. RBCs represent a good reporter for many tissues, including skin, especially due to their long lifetime in the human body, i.e., 120 days [105]. This lifetime is important also to observe changes in the membrane fatty acid composition during treatments. It is assumed that when the patient changes eating habits and uses appropriate supplementation, the fatty acid balance in cell membranes can change within 4 months, and this is also a good time frame to observe stable clinical improvements [106,107].

## 8. Conclusions

The importance of fatty acid balance for dermatological problems is very well recognized and reported in the literature. This suggests improving the use of diagnostic tools, such as the fatty acid-based membrane lipidomic analysis—using red blood cells—to modernize the protocols in skin diseases. It will allow for a personalized approach to the patient using a molecular tool with reliable and proven significance for the assessment of health status. Information about the lipid profile of individual patients may be important in assessing the causes of atopic dermatitis in population studies. By knowing the fatty acid profile of individual patients, it is possible to develop a strategy for keeping or restoring a healthy balance, favorable for the structural and functional roles of the membrane lipids. It can be accomplished by altering the patient’s eating habits and introducing a properly balanced diet together with specially selected supplements. A low SFA and TFA content and an appropriate ratio of omega-3 to omega-6 and MUFA to PUFA may be the proper treatment direction to prevent atopic disease and also ameliorate the inflammatory symptoms.

## Figures and Tables

**Table 1:** Prevalence of atopic dermatitis in adults in different countries.

Country	1-Year Prevalence of AD in Adults	Lifetime Prevalence of AD in Adults	Ref.
United States	4.9%	-	[24]
Canada	3.5%	-	[24]
Finland	10.1%	21.9%	[25]
Germany	2.2%	2.0–4.0%	[26]
France	-	3.6%	[27]
Italy	8.1%	4.7%	[27,28]
Japan	3.0%	3.3%	[29]

**Table 2:** The main food sources of the fatty acids that influence atopic dermatitis [60].

Fatty Acid	Food Source *
SFA	meat, dairy products, fast food
Palmitic acid (C16:0)	meat, cheese, butter, palm oil
MUFA	vegetable oils, dry fruits, olive, olive oil, butter
Oleic acid (C18:1, *n*-9)	vegetable oils, e.g., olive oil, margarine, avocado, olives
PUFA omega-6 and omega-3	fatty fish, algae, flax seed and oil, chia seeds, hemp seeds and oil, walnuts, almonds, hazelnuts, and other nuts
Linoleic acid (C18:2, *n*-6)	sunflower, corn, and safflower oils
Alpha-linolenic acid (C18:3, *n*-3)	flaxseeds and oil, canola oil, chia seed, purslane, algae
Gamma-linolenic acid (C18:3, *n*-6)	primrose and oil, borage and oil, blackcurrant and oil
Arachidonic acid (ARA, C20:4, *n*-6)	eggs, meat
Eicosapentaenoic acid (EPA, C20:5, *n*-3)	fish and fish oil, algae
Docosahexaenoic acid (DHA, C22:6, *n*-3)	fish oil and algae
Trans fatty acids	meat, milk, partially hydrogenated fats in foods, e.g., margarine

* For detailed composition, see: https://www.bda-ieo.it, accessed on 9 June 2023.

**Table 3:** Main fatty acids from human specimens or food sources involved in dermatological affections and clinical correlations.

Fatty Acid Change/Detection Site or Natural Source	Dermatological Affection	Clinical Correlation	Ref.
SFA/foods	Atopic dermatitis	Evaluated in mothers’ diet/high atopic sensitization in infants	[63,64,65]
Omega-6/omega-3 PUFA unbalance/ plasma phospholipids	Atopic dermatitis	Measured in mothers and infants—increased risk in the first year of life	[3]
MUFA increase/skin	Atopic dermatitis	Detected in atopic dermatitis lesions	[65,66,67]
MUFA increase/sebum	Atopic and seborrheic dermatitis	Disruption of the stratum corneum barrier	[68,69]
GLA/primrose or borage oil	Atopic dermatitis	Amelioration of dermatological symptoms	[6,70,79,80]
DGLA decrease/blood	Atopic dermatitis	Desaturase enzyme deficiency signature	[78]
GLA deficiency	Atopic dermatitis in infants	Amelioration of skin affection	[84]
PUFA omega-6 increase/blood cells and plasma	Eczema, atopic dermatitis	Inflammatory signature	[9,59,71,72,73,86]
Trans fatty acids/red blood cells, T-lymphocytes	Eczema, atopic dermatitis	Radical stress signature. Intakes of processed foods	[89,90,91]

Abbreviations: MUFA: monounsaturated fatty acid; GLA: gamma linolenic acid; PUFA: polyunsaturated fatty acid; DGLA: dihomo gamma linolenic acid.

## Data Availability

Not applicable.

## References

[1] Bhattacharya N., Sato W.J., Kelly A., Ganguli-Indra G., Indra A.K. (2019). Epidermal Lipids: Key Mediators of Atopic Dermatitis Pathogenesis. Trends. Mol. Med..

[2] Tomczak H., Wróbel J., Jenerowicz D., Sadowska-Przytocka A., Wachal M., Adamski Z., Czarnecka-Operacz M.M. (2019). The Role of Staphylococcus Aureus in Atopic Dermatitis: Microbiological and Immunological Implications. Postepy Dermatol. Allergol..

[3] Barman M., Stråvik M., Broberg K., Sandin A., Wold A.E., Sandberg A.-S. (2021). Proportions of Polyunsaturated Fatty Acids in Umbilical Cord Blood at Birth Are Related to Atopic Eczema Development in the First Year of Life. Nutrients.

[4] Sala-Vila A., Miles E.A., Calder P.C. (2008). Fatty Acid Composition Abnormalities in Atopic Disease: Evidence Explored and Role in the Disease Process Examined. Clin. Exp. Allergy.

[5] Miles E.A., Calder P.C. (2017). Can Early Omega-3 Fatty Acid Exposure Reduce Risk of Childhood Allergic Disease?. Nutrients.

[6] Kaźmierska A., Bolesławska I., Polańska A., Dańczak-Pazdrowska A., Jagielski P., Drzymała-Czyż S., Adamski Z., Przysławski J. (2022). Effect of Evening Primrose Oil Supplementation on Selected Parameters of Skin Condition in a Group of Patients Treated with Isotretinoin—A Randomized Double-Blind Trial. Nutrients.

[7] Spector A.A., Kiser R.E., Denning G.M., Koh S.-W., DeBault L.E. (1979). Modification of the Fatty Acid Composition of Cultured Human Fibroblasts. J. Lipid Res..

[8] de Carvalho C.C.C.R., Caramujo M.J. (2018). The Various Roles of Fatty Acids. Molecules.

[9] Ferreri C., Chatgilialoglu C. (2015). Membrane Lipidomics for Personalized Health.

[10] Yang M., Zhou M., Song L. (2020). A Review of Fatty Acids Influencing Skin Condition. J. Cosmet. Dermatol..

[11] Cui L., Jia Y., Cheng Z., Gao Y., Zhang G., Li J., He C. (2016). Advancements in the Maintenance of Skin Barrier/Skin Lipid Composition and the Involvement of Metabolic Enzymes. J. Cosmet. Dermatol..

[12] Coomans de Brachène A., Dif N., de Rocca Serra A., Bonnineau C., Velghe A.I., Larondelle Y., Tyteca D., Demoulin J. (2017). PDGF-induced Fibroblast Growth Requires Monounsaturated Fatty Acid Production by Stearoyl-CoA Desaturase. FEBS Open Bio..

[13] Alatibi K.I., Hagenbuchner J., Wehbe Z., Karall D., Ausserlechner M.J., Vockley J., Spiekerkoetter U., Grünert S.C., Tucci S. (2021). Different Lipid Signature in Fibroblasts of Long-Chain Fatty Acid Oxidation Disorders. Cells.

[14] Kendall A.C., Pilkington S.M., Massey K.A., Sassano G., Rhodes L.E., Nicolaou A. (2015). Distribution of Bioactive Lipid Mediators in Human Skin. J. Invest. Dermatol..

[15] Kendall A.C., Nicolaou A. (2013). Bioactive Lipid Mediators in Skin Inflammation and Immunity. Prog Lipid Res.

[16] Williams H., Flohr C. (2006). How Epidemiology Has Challenged 3 Prevailing Concepts about Atopic Dermatitis. J. Allergy Clin. Immunol..

[17] Williams H., Stewart A., von Mutius E., Cookson W., Anderson H.R., of Asthma I.S. (2008). Is Eczema Really on the Increase Worldwide?. J. Allergy Clin. Immunol..

[18] Schäfer T., Vieluf D., Behrendt H., Kramer U., Ring J. (1996). Atopic Eczema and Other Manifestations of Atopy: Results of a Study in East and West Germany. Allergy.

[19] Strachan D.P. (1989). Hay Fever, Hygiene, and Household Size. BMJ Br. Med. J..

[20] von Kobyletzki L.B., Bornehag C.G., Breeze E., Larsson M., Lindström C.B., Svensson Å. (2014). Factors Associated with Remission of Eczema in Children: A Population-Based Follow-up Study. Acta Derm. Venereol..

[21] Kim J., Kim B.E., Leung D.Y.M. (2019). Pathophysiology of Atopic Dermatitis: Clinical Implications. Allergy Asthma Proc..

[22] Ruzicka T., Ring J., Przybilla B. (2013). Handbook of Atopic Eczema.

[23] Raimondo A., Lembo S. (2021). Atopic Dermatitis: Epidemiology and Clinical Phenotypes. Dermatol Pr. Concept.

[24] Odhiambo J.A., Williams H.C., Clayton T.O., Robertson C.F., Asher M.I., Group I.P.T.S. (2009). Global Variations in Prevalence of Eczema Symptoms in Children from ISAAC Phase Three. J. Allergy Clin. Immunol..

[25] Kiiski V., Salava A., Susitaival P., Barnhill S., Remitz A., Heliovaara M. (2022). Atopic Dermatitis in Adults: A Population-based Study in Finland. Int. J. Dermatol..

[26] Mohr N., Naatz M., Zeervi L., Langenbruch A., Bieber T., Werfel T., Wollenberg A., Augustin M. (2021). Cost-of-illness of Atopic Dermatitis in Germany: Data from Dermatology Routine Care. J. Eur. Acad. Dermatol. Venereol..

[27] Kleyn C.E., Barbarot S., Reed C., Losi S., von Arx L.-B., Robert C., Anderson P., Grond S., Costanzo A. (2022). Burden of Moderate to Severe Atopic Dermatitis in Adults from France, Italy, and the UK: Patient-Reported Outcomes and Treatment Patterns. Dermatol. Ther..

[28] Naldi L., Colombo P., Placchesi E.B., Piccitto R., Chatenoud L., la Vecchia C. (2004). Study Design and Preliminary Results from the Pilot Phase of the PraKtis Study: Self-Reported Diagnoses of Selected Skin Diseases in a Representative Sample of the Italian Population. Dermatology.

[29] Barbarot S., Auziere S., Gadkari A., Girolomoni G., Puig L., Simpson E.L., Margolis D.J., de Bruin-Weller M., Eckert L. (2018). Epidemiology of Atopic Dermatitis in Adults: Results from an International Survey. Allergy.

[30] Nutten S. (2015). Atopic Dermatitis: Global Epidemiology and Risk Factors. Ann. Nutr. Metab..

[31] Weidinger S., Beck L.A., Bieber T., Kabashima K., Irvine A.D. (2018). Atopic dermatitis. Nat. Rev. Dis. Primers..

[32] Kim J.H., Lee S.W., Yon D.K., Ha E.K., Jee H.M., Sung M., Sim H.J., Yoon J.W., Choi S., Shin Y.H. (2021). Association of Serum Lipid Parameters with the SCORAD Index and Onset of Atopic Dermatitis in Children. Pediatr. Allergy Immunol..

[33] Chisaguano A.M., Montes R., Pérez-Berezo T., Castellote A.I., Guerendiain M., Bustamante M., Morales E., García-Esteban R., Sunyer J., Franch À. (2013). Gene Expression of Desaturase (FADS1 and FADS2) and Elongase (ELOVL5) Enzymes in Peripheral Blood: Association with Polyunsaturated Fatty Acid Levels and Atopic Eczema in 4-Year-Old Children. PLoS ONE.

[34] Al-Shobaili H.A., Ahmed A.A., Alnomair N., Alobead Z.A., Rasheed Z. (2016). Molecular Genetic of Atopic Dermatitis: An Update. Int. J. Health Sci. (Qassim).

[35] Nedoszytko B., Reszka E., Gutowska-Owsiak D., Trzeciak M., Lange M., Jarczak J., Niedoszytko M., Jablonska E., Romantowski J., Strapagiel D. (2020). Genetic and Epigenetic Aspects of Atopic Dermatitis. Int. J. Mol. Sci..

[36] Margolis D.J., Apter A.J., Gupta J., Hoffstad O., Papadopoulos M., Campbell L.E., Sandilands A., McLean W.H.I., Rebbeck T.R., Mitra N. (2012). The Persistence of Atopic Dermatitis and Filaggrin (FLG) Mutations in a US Longitudinal Cohort. J. Allergy Clin. Immunol..

[37] Hsu C., Akiyama M., Nemoto-Hasebe I., Nomura T., Sandilands A., Chao S., Lee J., Sheu H., McLean W.H.I., Shimizu H. (2009). Analysis of Taiwanese Ichthyosis Vulgaris Families Further Demonstrates Differences in FLG Mutations between European and Asian Populations. Br. J. Dermatol..

[38] Palmer C.N.A., Irvine A.D., Terron-Kwiatkowski A., Zhao Y., Liao H., Lee S.P., Goudie D.R., Sandilands A., Campbell L.E., Smith F.J.D. (2006). Common Loss-of-Function Variants of the Epidermal Barrier Protein Filaggrin Are a Major Predisposing Factor for Atopic Dermatitis. Nat. Genet.

[39] Watson W., Kapur S. (2011). Atopic Dermatitis. Allergy, Asthma & Clinical Immunology. Eczema (Atopic Dermat.) Treat..

[40] Martin M.J., Estravís M., García-Sánchez A., Dávila I., Isidoro-García M., Sanz C. (2020). Genetics and Epigenetics of Atopic Dermatitis: An Updated Systematic Review. Genes.

[41] Stemmler S., Hoffjan S. (2016). Trying to Understand the Genetics of Atopic Dermatitis. Mol. Cell Probes.

[42] Rzehak P., Heinrich J., Klopp N., Schaeffer L., Hoff S., Wolfram G., Illig T., Linseisen J. (2008). Evidence for an Association between Genetic Variants of the Fatty Acid Desaturase 1 Fatty Acid Desaturase 2 (FADS1 FADS2) Gene Cluster and the Fatty Acid Composition of Erythrocyte Membranes. Br. J. Nutr..

[43] Rzehak P., Thijs C., Standl M., Mommers M., Glaser C., Jansen E., Klopp N., Koppelman G.H., Singmann P., Postma D.S. (2010). Variants of the FADS1 FADS2 Gene Cluster, Blood Levels of Polyunsaturated Fatty Acids and Eczema in Children within the First 2 Years of Life. PLoS ONE.

[44] Tanjung C., Rzehak P., Sudoyo H., Mansyur M., Munasir Z., Immanuel S., Irawan R., Reischl E., Demmelmair H., Hadinegoro S.R. (2018). The Association of Fatty Acid Desaturase Gene Polymorphisms on Long-Chain Polyunsaturated Fatty Acid Composition in Indonesian Infants. Am. J. Clin. Nutr..

[45] Lattka E., Klopp N., Demmelmair H., Klingler M., Heinrich J., Koletzko B. (2012). Genetic Variations in Polyunsaturated Fatty Acid Metabolism–Implications for Child Health?. Ann. Nutr. Metab..

[46] Standl M., Sausenthaler S., Lattka E., Koletzko S., Bauer C., Wichmann H., von Berg A., Berdel D., Krämer U., Schaaf B. (2011). FADS Gene Variants Modulate the Effect of Dietary Fatty Acid Intake on Allergic Diseases in Children. Clini. Exp. Allergy.

[47] Conway M.C., McSorley E.M., Mulhern M.S., Strain J.J., van Wijngaarden E., Yeates A.J. (2020). Influence of Fatty Acid Desaturase (FADS) Genotype on Maternal and Child Polyunsaturated Fatty Acids (PUFA) Status and Child Health Outcomes: A Systematic Review. Nutr. Rev..

[48] Simon D., Eng P.A., Borelli S., Kägi R., Zimmermann C., Zahner C., Drewe J., Hess L., Ferrari G., Lautenschlager S. (2014). Gamma-Linolenic Acid Levels Correlate with Clinical Efficacy of Evening Primrose Oil in Patients with Atopic Dermatitis. Adv. Ther..

[49] Schaeffer L., Gohlke H., Müller M., Heid I.M., Palmer L.J., Kompauer I., Demmelmair H., Illig T., Koletzko B., Heinrich J. (2006). Common Genetic Variants of the FADS1 FADS2 Gene Cluster and Their Reconstructed Haplotypes Are Associated with the Fatty Acid Composition in Phospholipids. Hum. Mol. Genet..

[50] Singmann P., Rzehak P., Berdel D., Wichmann H.E., Heinrich J. (2010). No Association between FADS Polymorphisms and Atopic Diseases in Children from the GINI and LISA Birth Cohorts. Allergy.

[51] Standl M., Sausenthaler S., Lattka E., Koletzko S., Bauer C., Wichmann H., Von Berg A., Berdel D., Krämer U., Schaaf B. (2012). FADS Gene Cluster Modulates the Effect of Breastfeeding on Asthma. Results from the GINIplus and LISAplus Studies. Allergy.

[52] Barman M., Nilsson S., Torinsson Naluai Å., Sandin A., Wold A.E., Sandberg A.-S. (2015). Single Nucleotide Polymorphisms in the FADS Gene Cluster but Not the ELOVL2 Gene Are Associated with Serum Polyunsaturated Fatty Acid Composition and Development of Allergy (in a Swedish Birth Cohort). Nutrients.

[53] Totté J.E.E., van der Feltz W.T., Hennekam M., van Belkum A., van Zuuren E.J., Pasmans S. (2016). Prevalence and Odds of Staphylococcus Aureus Carriage in Atopic Dermatitis: A Systematic Review and Meta-analysis. Br. J. Dermatol..

[54] Incorvaia C., Frati F., Verna N., D’Alò S., Motolese A., Pucci S. (2008). Allergy and the Skin. Clin. Exp. Immunol..

[55] Manti S., Leonardi S., Panasiti I., Arrigo T., Salpietro C., Cuppari C. (2017). Serum IL-10, IL-17 and IL-23 Levels as “Bioumoral Bridges” between Dyslipidemia and Atopy. Cytokine.

[56] Balić A., Vlašić D., Žužul K., Marinović B., Bukvić Mokos Z. (2020). Omega-3 versus Omega-6 Polyunsaturated Fatty Acids in the Prevention and Treatment of Inflammatory Skin Diseases. Int. J. Mol. Sci..

[57] Venter C., Meyer R.W., Nwaru B.I., Roduit C., Untersmayr E., Adel-Patient K., Agache I., Agostoni C., Akdis C.A., Bischoff S.C. (2019). EAACI Position Paper: Influence of Dietary Fatty Acids on Asthma, Food Allergy, and Atopic Dermatitis. Allergy.

[58] Venter C., Eyerich S., Sarin T., Klatt K.C. (2020). Nutrition and the Immune System: A Complicated Tango. Nutrients.

[59] Horrobin D.F. (2000). Essential Fatty Acid Metabolism and Its Modification in Atopic Eczema. Am. J. Clin. Nutr..

[60] Kaur N., Chugh V., Gupta A.K. (2014). Essential Fatty Acids as Functional Components of Foods-a Review. J. Food Sci. Technol..

[61] Kang C.-M., Chiang B.-L., Wang L.-C. (2021). Maternal Nutritional Status and Development of Atopic Dermatitis in Their Offspring. Clin. Rev. Allergy Immunol..

[62] Hoppu U., Kalliomäki M., Isolauri E. (2000). Maternal Diet Rich in Saturated Fat during Breastfeeding Is Associated with Atopic Sensitization of the Infant. Eur. J. Clin. Nutr..

[63] Ellwood P., Asher M.I., García-Marcos L., Williams H., Keil U., Robertson C., Nagel G., Group I.P.I.I.I.S. (2013). Do Fast Foods Cause Asthma, Rhinoconjunctivitis and Eczema? Global Findings from the International Study of Asthma and Allergies in Childhood (ISAAC) Phase Three. Thorax.

[64] Wang C.S., Wang J., Zhang X., Zhang L., Zhang H.P., Wang L., Wood L.G., Wang G. (2018). Is the Consumption of Fast Foods Associated with Asthma or Other Allergic Diseases?. Respirology.

[65] Schäfer L., Kragballe K. (1991). Abnormalities in Epidermal Lipid Metabolism in Patients with Atopic Dermatitis. J. Invest. Dermatol..

[66] Trak-Fellermeier M.A., Brasche S., Winkler G., Koletzko B., Heinrich J. (2004). Food and Fatty Acid Intake and Atopic Disease in Adults. Eur. Respir. J..

[67] Heinrich J., Hölscher B., Bolte G., Winkler G. (2001). Allergic Sensitization and Diet: Ecological Analysis in Selected European Cities. Eur. Respir. J..

[68] Mojumdar E.H., Helder R.W.J., Gooris G.S., Bouwstra J.A. (2014). Monounsaturated Fatty Acids Reduce the Barrier of Stratum Corneum Lipid Membranes by Enhancing the Formation of a Hexagonal Lateral Packing. Langmuir.

[69] Agrawala K., Hassoun L.A., Foolad N., Borkowski K., Pedersen T.L., Sivamani R.K., Newman J.W. (2018). Effects of Atopic Dermatitis and Gender on Sebum Lipid Mediator and Fatty Acid Profiles. Prostaglandins Leukot Essent Fat. Acids.

[70] Calder P.C. (2012). Long-Chain Fatty Acids and Inflammation. Proc. Nutr. Soc..

[71] Flohr C., Mann J. (2014). New Insights into the Epidemiology of Childhood Atopic Dermatitis. Allergy.

[72] Devereux G., Seaton A. (2005). Diet as a Risk Factor for Atopy and Asthma. J. Allergy Clin. Immunol..

[73] Schäfer L., Kragballe K. (1991). Supplementation with Evening Primrose Oil in Atopic Dermatitis: Effect on Fatty Acids in Neutrophils and Epidermis. Lipids.

[74] Chatgilialoglu C., Ferreri C., Melchiorre M., Sansone A., Torreggiani A. (2014). Lipid Geometrical Isomerism: From Chemistry to Biology and Diagnostics. Chem. Rev..

[75] Waehler R. (2021). Fatty Acids: Facts vs. Fiction. Int. J. Vitam. Nutr. Res..

[76] Calder P.C., Grimble R.F. (2002). Polyunsaturated Fatty Acids, Inflammation and Immunity. Eur. J. Clin. Nutr/.

[77] Calder P.C. (2007). Dietary Arachidonic Acid: Harmful, Harmless or Helpful?. Br. J. Nutr..

[78] Kawashima H. (2019). Intake of Arachidonic Acid-Containing Lipids in Adult Humans: Dietary Surveys and Clinical Trials. Lipids. Health Dis..

[79] Foster R.H., Hardy G., Alany R.G. (2010). Borage Oil in the Treatment of Atopic Dermatitis. Nutrition.

[80] Barham J.B., Edens M.B., Fonteh A.N., Johnson M.M., Easter L., Chilton F.H. (2000). Addition of Eicosapentaenoic Acid to γ-Linolenic Acid–Supplemented Diets Prevents Serum Arachidonic Acid Accumulation in Humans. J. Nutr..

[81] Amagai Y., Oida K., Matsuda A., Jung K., Kakutani S., Tanaka T., Matsuda K., Jang H., Ahn G., Xia Y. (2015). Dihomo-γ-Linolenic Acid Prevents the Development of Atopic Dermatitis through Prostaglandin D1 Production in NC/Tnd Mice. J. Dermatol. Sci..

[82] Sertznig P., Reichrath J. (2011). Peroxisome Proliferator-Activated Receptors (PPARs) in Dermatology: Challenge and Promise. Dermatoendocrinol.

[83] Blunder S., Pavel P., Minzaghi D., Dubrac S. (2021). PPARdelta in Affected Atopic Dermatitis and Psoriasis: A Possible Role in Metabolic Reprograming. Int. J. Mol. Sci..

[84] Nagano N., Okada T., Kayama K., Hosono S., Kitamura Y., Takahashi S. (2016). Delta-6 Desaturase Activity during the First Year of Life in Preterm Infants. Prostaglandins Leukot. Essent. Fatty Acids.

[85] Töröcsik D., Weise C., Gericke J., Szegedi A., Lucas R., Mihaly J., Worm M., Rühl R. (2019). Transcriptomic and Lipidomic Profiling of Eicosanoid/Docosanoid Signalling in Affected and Non-affected Skin of Human Atopic Dermatitis Patients. Exp. Dermatol..

[86] Lindskou R., Hølmer G. (1992). Polyunsaturated Fatty Acids in Plasma, Red Blood Cells and Mononuclear Cell Phospholipids of Patients with Atopic Dermatitis. Allergy.

[87] Nagel G., Linseisen J. (2005). Dietary Intake of Fatty Acids, Antioxidants and Selected Food Groups and Asthma in Adults. Eur. J. Clin. Nutr..

[88] Welland S.K., Mutius E., Husing A. (1999). Intake of Trans Fatty Acids and Prevalence of Childhood Asthma and Allergies in Europe. Lancet.

[89] Ferreri C., Angelini F., Chatgilialoglu C., Dellonte S., Moschese V., Rossi P., Chini L. (2005). Trans Fatty Acids and Atopic Eczema/Dermatitis Syndrome: The Relationship with a Free Radical Cis-Trans Isomerization of Membrane Lipids. Lipids.

[90] Hung W.-L., Hwang L.S., Shahidi F., Pan M.-H., Wang Y., Ho C.-T. (2016). Endogenous Formation of Trans Fatty Acids: Health Implications and Potential Dietary Intervention. J. Funct. Foods.

[91] Kwon Y. (2016). Effect of Trans–Fatty Acids on Lipid Metabolism: Mechanisms for Their Adverse Health Effects. Food Rev. Int..

[92] Rosenthal M.D., Whitehurst M.C. (1983). Selective Effects of Isomeric Cis and Trans Fatty Acids on Fatty Acyl Δ9 and Δ6 Desaturation by Human Skin Fibroblasts. Biochim. Biophys. Acta (BBA)-Lipids Lipid Metab..

[93] Sakai T., Ire A.V., Matoba T., Yamamoto S. (2009). Dietary Trans Fatty Acids Suppress the Development of Spontaneous Atopic-like Dermatitis in NC/Nga Mice. J. Nutr. Sci. Vitaminol..

[94] Lopez-Garcia E., Schulze M.B., Meigs J.B., Manson J.E., Rifai N., Stampfer M.J., Willett W.C., Hu F.B. (2005). Consumption of Trans Fatty Acids Is Related to Plasma Biomarkers of Inflammation and Endothelial Dysfunction. J. Nutr..

[95] Mozaffarian D., Rimm E.B., King I.B., Lawler R.L., McDonald G.B., Levy W.C. (2004). Trans Fatty Acids and Systemic Inflammation in Heart Failure. Am. J. Clin. Nutr..

[96] Lin J.-Y., Ma L.-J., Yuan J.-P., Yu P., Bai B.-X. (2023). Causal Effects of Fatty Acids on Atopic Dermatitis: A Mendelian Randomization Study. Front. Nutr..

[97] Thomsen B.J., Chow E.Y., Sapijaszko M.J. (2020). The Potential Uses of Omega-3 Fatty Acids in Dermatology: A Review. J. Cutan. Med. Surg..

[98] Nicolson G.L., Ash M.E. (2014). Lipid Replacement Therapy: A Natural Medicine Approach to Replacing Damaged Lipids in Cellular Membranes and Organelles and Restoring Function. Biochim. Et Biophys. Acta (BBA)-Biomembr..

[99] Liu R.-L., Zhang J., Mou Z.-L., Hao S.-L., Zhang Z.-Q. (2012). Microwave-Assisted One-Step Extraction-Derivatization for Rapid Analysis of Fatty Acids Profile in Herbal Medicine by Gas Chromatography-Mass Spectrometry. Analyst.

[100] Gallego S.F., Hermansson M., Liebisch G., Hodson L., Ejsing C.S. (2019). Total Fatty Acid Analysis of Human Blood Samples in One Minute by High-Resolution Mass Spectrometry. Biomolecules.

[101] Sender R., Fuchs S., Milo R. (2016). Revised Estimates for the Number of Human and Bacteria Cells in the Body. PLoS Biol..

[102] Brenna J.T., Plourde M., Stark K.D., Jones P.J., Lin Y.-H. (2018). Best Practices for the Design, Laboratory Analysis, and Reporting of Trials Involving Fatty Acids. Am. J. Clin. Nutr..

[103] Paul J.-L., Sall N.-D., Soni T., Poignet J.-L., Lindenbaum A., Man N.-K., Moatti N., Raichvarg D. (1993). Lipid Peroxidation Abnormalities in Hemodialyzed Patients. Nephron.

[104] Nowak E., Wyrwicz G., Smoleński O., Spodaryk K. (1999). Rheological Properties of Red Blood Cells (Including Reticulocytes) in Patients with Chronic Renal Disease. Clin. Hemorheol. Microcirc..

[105] Hodson L., Skeaff C.M., Fielding B.A. (2008). Fatty Acid Composition of Adipose Tissue and Blood in Humans and Its Use as a Biomarker of Dietary Intake. Prog. Lipid Res..

[106] Ferreri C., Masi A., Sansone A., Giacometti G., Larocca A.V., Menounou G., Scanferlato R., Tortorella S., Rota D., Conti M. (2017). Fatty Acids in Membranes as Homeostatic, Metabolic and Nutritional Biomarkers: Recent Advancements in Analytics and Diagnostics. Diagnostics.

[107] Ferreri C., Chatgilialoglu C. (2012). Role of Fatty Acid-Based Functional Lipidomics in the Development of Molecular Diagnostic Tools. Expert Rev. Mol. Diagn..

